# The ADP receptor P2Y_1 _is necessary for normal thermal sensitivity in cutaneous polymodal nociceptors

**DOI:** 10.1186/1744-8069-7-13

**Published:** 2011-02-10

**Authors:** Derek C Molliver, Kristofer K Rau, Sabrina L McIlwrath, Michael P Jankowski, H Richard Koerber

**Affiliations:** 1 Department of Medicine, University of Pittsburgh, Pittsburgh, PA, USA; 2 Department of Neurobiology, University of Pittsburgh, Pittsburgh, PA, USA

## Abstract

**Background:**

P2Y_1 _is a member of the P2Y family of G protein-coupled nucleotide receptors expressed in peripheral sensory neurons. Using ratiometric calcium imaging of isolated dorsal root ganglion neurons, we found that the majority of neurons responding to adenosine diphosphate, the preferred endogenous ligand, bound the lectin IB4 and expressed the ATP-gated ion channel P2X_3_. These neurons represent the majority of epidermal afferents in hairy skin, and are predominantly C-fiber polymodal nociceptors (CPMs), responding to mechanical stimulation, heat and in some cases cold.

**Results:**

To characterize the function of P2Y_1 _in cutaneous afferents, intracellular recordings from sensory neuron somata were made using an *ex vivo *preparation in which the hindlimb skin, saphenous nerve, DRG and spinal cord were dissected in continuum, and cutaneous receptive fields characterized using digitally-controlled mechanical and thermal stimuli in male wild type mice. In P2Y_1_-/- mice, CPMs showed a striking increase in mean heat threshold and a decrease in mean peak firing rate during a thermal ramp from 31-52°C. A similar change in mean cold threshold was also observed. Interestingly, mechanical testing of CPMs revealed no significant differences between P2Y_1_-/- and WT mice.

**Conclusions:**

These results strongly suggest that P2Y_1 _is required for normal thermal signaling in cutaneous sensory afferents. Furthermore, they suggest that nucleotides released from peripheral tissues play a critical role in the transduction of thermal stimuli in some fiber types.

## Background

During tissue injury, elevated concentrations of extracellular adenosine triphosphate (ATP) contribute to the activation of nociceptive sensory afferents, resulting in hyperalgesia [1]. Injection of ATP into human skin produces burning pain [2], and injection into rat plantar skin generates a dose-dependent nocifensive foot withdrawal response [3]. This activation of sensory fibers occurs through the binding of ATP onto two families of receptors: P2X ionotropic cation receptors and P2Y metabotropic G-protein coupled receptors (GPCRs) [4-9]. While a significant amount of research has examined the seven known subtypes of P2X receptors P2X_1-7 _[10-12] and their involvement in nociception [13-15], the role of P2Y receptors in nociception remains an area of active investigation.

Thus far, there are eight known members of the P2Y family (P2Y_1, 2, 4, 6, 11, 12, 13 and 14_). Of these receptors, the G_q_-coupled P2Y_1_, P2Y_2_, and the G_i/o_-coupled P2Y_12_, P2Y_13_, and P2Y_14 _are expressed in high levels in sensory neurons of dorsal root ganglia (DRG) [16-21]. G protein coupling has been described in numerous cell types [4]; in isolated DRG neurons, we have confirmed that P2Y_1 _is coupled to release of intracellular Ca^++ ^stores, whereas P2Y_12-14 _are coupled to inhibition of voltage-dependent Ca^++ ^channels [22,21]. P2Y_4 _and P2Y_6 _are also expressed in DRG, but at lower levels [18,20,23]. While reports of P2Y_1 _distribution in DRG vary widely [8,16,19,20,24-26], several studies have reported that P2Y_1 _is expressed in a subpopulation of sensory afferents: small diameter neurons that contain P2X_3_, bind the isolectin B4 (IB4) from *Griffonia simplicifolia*, and lack the capsaicin-, heat-, and proton-sensitive transient receptor potential vanilloid receptor-1 (TRPV1). This subpopulation represents the majority of cutaneous afferents in mouse.

The consequences of P2Y_1 _activation in nociceptors are also controversial: previous *in vitro *studies have shown that P2Y_1 _receptor activation has inhibitory effects on currents generated by N-type calcium channels (Ca_v_2.2) [25] and P2X_3 _receptors [26,27], which can decrease the release of nociceptive transmitters in the spinal cord [8]. In contrast, P2Y_1 _receptors have also been implicated in responses to low-threshold mechanical stimuli in a *Xenopus *oocyte expression system [16]. Supporting a role for P2Y_1 _in nociception, injection of the P2Y_1 _agonist adenosine diphosphate (ADP) into the hindpaw caused heat hyperalgesia in wildtype but not in P2Y_1 _knockout, mice [21]. While these studies have suggested multiple functions for the P2Y_1 _receptor in sensory perception, it has not been determined which neuronal cell type(s) transduce P2Y_1_-mediated signals from peripheral receptive fields. Furthermore, the impact of P2Y1 signaling on the transduction of nociceptive stimuli has not been resolved.

In the present study, we identified and characterized a population of cutaneous afferents that express P2Y_1 _using Ca^2+ ^imaging and sharp electrode electrophysiology in an *ex vivo *skin/nerve/DRG/spinal cord preparation. We found that the large majority of IB4-binding neurons respond to the preferred P2Y_1 _agonist ADP with an increase in intracellular Ca^2+^. Electrophysiological analysis revealed that these neurons were polymodal in function, responding to mechanical and heat stimuli, as well as to cold stimuli in some cases. Deletion of P2Y_1 _resulted in a significantly reduced excitability of these sensory afferents, which consisted of a decreased sensitivity to both warming and cooling.

## Results

### Identification of ADP Responses in IB4-Binding Neurons

**Figure 1:**
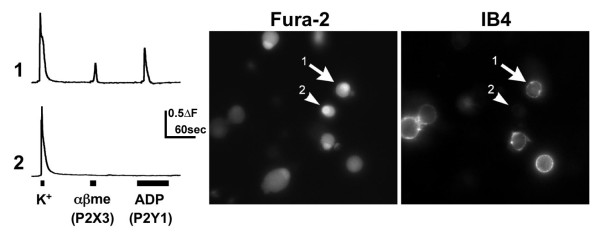
**ADP-evoked Ca^2+ ^transients in IB4-positive neurons**. Examples of isolated neurons characterized by Fura-2 Ca^2+ ^imaging and IB4-binding. Neurons were labeled with IB4 prior to use, photographed and then tested by Ca^2+ ^imaging for responsiveness to 50 mM K^+ ^to identify healthy neurons, then ADP (100 μM). Some of these neurons were also tested with α,β-me ATP, a selective agonist for P2X_3_-containing ion channels (100 μM, αβ-me ATP). Two representative traces are shown from a single field: **Cell 1 **responded to αβ-me ATP and ADP and bound IB4. **Cell 2 **did not respond to αβ-me ATP or ADP and did not bind IB4. Scale is shown as the change in relative fluorescence units (ΔF) versus time (sec).

Several previous studies have reported that P2Y_1 _is preferentially expressed in the IB4-binding population of small-diameter sensory neurons [24,28]. These neurons represent the majority of cutaneous C-fiber afferents. To confirm these previous histological results with functional data, we tested the ability of IB4-binding neurons to show functional responses to the P2Y_1 _agonist ADP (100 μM), as well as to the P2X_3_, P2X_2/3 _agonist α,β-me ATP (Figure 1). 73.5% (86/117) of IB4-binding neurons showed a transient increase in intracellular Ca^2+ ^in response to ADP. The large majority of ADP-responsive neurons (77%, 47/61) also responded to α,β-me ATP, indicating expression of P2X_3_. These results indicated widespread expression of functional P2Y_1 _receptors in IB4-binding, P2X_3_-expressing DRG neurons in acute cell culture.

### Classification and Distribution of Cutaneous Sensory Neurons

Neurons recorded in the *ex vivo *preparation were sorted into subgroups depending upon their conduction velocities (CV) and responses to mechanical and thermal stimuli. Neurons with a conduction velocity of <1.2 m/s were classified as C-fibers, and all others were classified as A-fibers [29,30].

Although we recorded from both A- and C-fibers, we focused our recording efforts primarily on C-fibers that were characterized as C-polymodal (CPM) in function, responding to mechanical and heat stimuli (CMH), and sometimes to cold stimuli as well (CMHC). We only examined enough A-fibers and other classes of C-fiber to verify that there were no significant differences in their biophysical characteristics or response properties to cutaneous stimuli (data not shown). Furthermore, while we occasionally observed mechanically and thermally unresponsive cells that were driven by the peripheral stimulating electrode, we only analyzed cells that had an identifiable cutaneous receptive field.

A total of 135 CPM fibers innervating hindlimb hairy skin via the saphenous nerve were intracellularly recorded and physiologically characterized from 18 WT (n = 69 cells) and 12 P2Y_1_-/- (n = 66 cells) adult male mice. Apart from the cold response in CMHC cells, there was no statistical difference between the CMH and CMHC groups for either strain, and therefore these populations were pooled together as CPM cells. It should be noted that not all of these cells were stained with Neurobiotin, either due to logistical reasons or loss of cell.

Biophysical data including CV (calculated as the distance between the stimulating and recording electrodes, divided by the spike latency between the stimulus pulse and the triggered action potential (AP)), AP amplitude and duration at half-amplitude were collected for each recorded cell (data not shown). No significant differences were observed in AP amplitude or half-amplitude width for either WT (2.23 ± 0.07 ms) or P2Y_1_-/- mice (2.13 ± 0.10 ms). However, the CV of CPMs in P2Y_1_-/- mice (0.59 ± 0.01 m/s) was slightly faster than the CV in WT mice (0.53 ± 0.01 ms; p < 0.05). This slight difference in conduction velocity would not be expected to be functionally significant.

### CPMs in P2Y_1_-/- mice have normal sensitivity to mechanical stimuli

**Figure 2:**
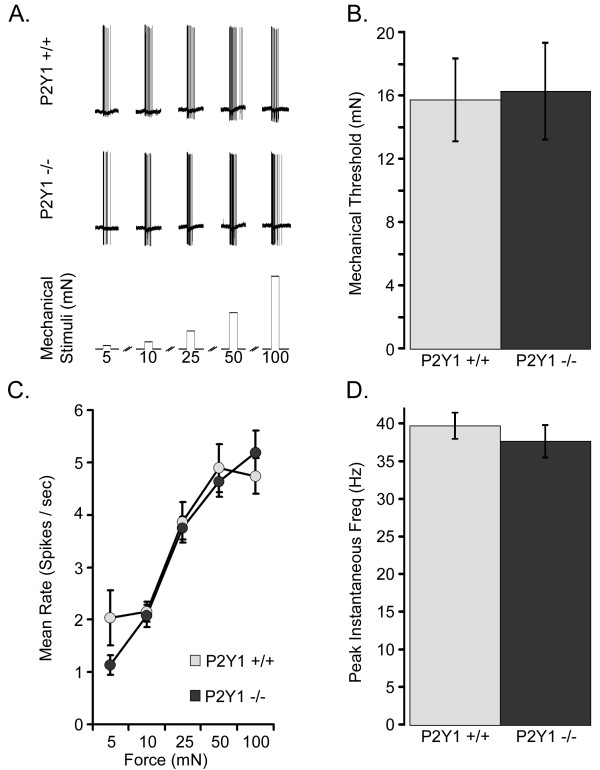
**CPMs in P2Y_1_-/- mice have normal mechanical sensitivity**. The response to 5, 10, 25, 50 and 100 mN mechanical stimuli is shown for CPM cells from WT and P2Y_1_-/- mice (**A**). Sharp electrode recordings from WT (grey) and P2Y_1_-/- (black) CPM cells revealed no significant difference between the strains in either the average mechanical threshold (**B**; mN), mean rate (**C**; spikes/sec; 5, 10, 25, 50 and 100 mN), or in the mean peak instantaneous frequency (**D**; Hz). Significance is indicated as p < 0.05 (*).

CPM fibers were tested for their response to mechanical stimuli (Figure 2A). No significant differences were observed in the mechanical response properties between WT and P2Y_1_-/- mice. These properties included average mechanical threshold (WT: 15.76 ± 2.62 mN vs. P2Y_1_-/-: 13.61 ± 2.48 mN; Figure 2B), peak instantaneous frequency (WT: 39.81 ± 1.78 Hz vs. P2Y_1_-/-: 39.60 ± 2.00 Hz; Figure 2D), and peak mean firing rate (WT: 5.52 ± 0.44 spikes/sec vs. P2Y_1_-/-: 5.04 ± 0.28 spikes/sec; data not shown). Similarly, the overall mean firing rates in response to 5 mN, 10 mN, 25 mN, 50 mN and 100 mN stimuli remained unchanged between mouse strains (Figure 2C).

### CPMs in P2Y_1_-/- strains have decreased heat and cold sensitivity

**Figure 3:**
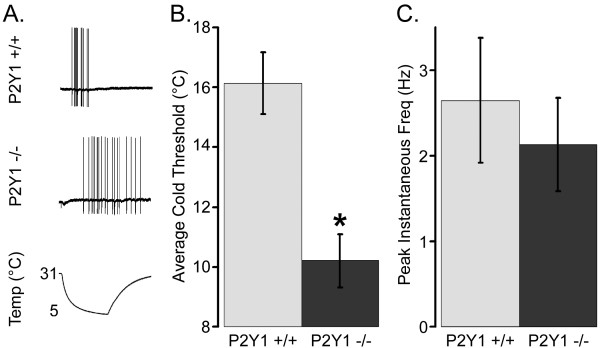
**CPMs in P2Y_1_-/- mice exhibit decreased cold sensitivity**. Sharp electrode recordings were made from WT and P2Y_1_-/- CPM afferents that were tested for their response to cold stimuli. A sample of the action potentials evoked by a cooling stimulus is shown (**A)**. Compared to WT strains (grey), P2Y_1_-/- (black) CPM cells had lower cold thresholds (**B**; °C), but maintained a similar peak instantaneous frequency (**C**; Hz). Significance is indicated as p < 0.05 (*).

CPM cells were tested for their response to cooling and warming stimuli. In CPM fibers that responded to cooling stimuli (Figure 3A), the average cold threshold in WT strains was significantly higher than those found in P2Y_1_-/- strains (Figure 3B). From a baseline bath temperature that was maintained at 31.0°C, a rapid decrease in temperature to approximately 4°C resulted in APs that were evoked at relatively higher temperatures in WT (16.14 ± 1.03°C) than in P2Y_1_-/- (10.62 ± 0.81°C; p < 0.05; Figure 3B). Maximum instantaneous frequency during cooling, however, was not significantly different between the strains (WT: 1.39 ± 0.29 Hz; P2Y_1_-/-; 1.44 ± 0.29 Hz; and P2Y_1_-/-: 1.44 ± 0.29 Hz; p < 0.05; Figure 3C).

**Figure 4:**
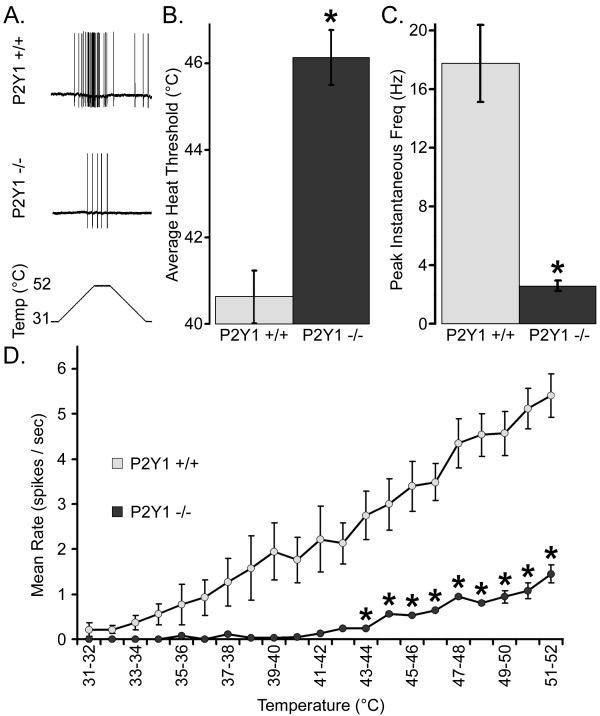
**CPMs in P2Y_1_-/- mice exhibit decreased heat sensitivity**. Sharp electrode recordings were made from WT and P2Y_1_-/- CPM neurons that were tested for their response to heat stimuli. A sample of the action potentials evoked by a heating ramp is shown (**A**). In comparison to WT mice (grey), the average thermal threshold of CPMs in mice lacking P2Y_1 _(black) showed decreased excitability in terms of a higher heat threshold (**B**; °C) and a lower peak instantaneous frequency (**C**; Hz). A heat ramp (**D**) from 31 to 52°C showed a significant reduction in the average spikes/second fired by CPMs from P2Y_1_-/- between the temperatures of 43-52°C. Significance is indicated as p < 0.05 (*).

During heating ramps from 31.0°C to 52.0°C (Figure 4A), CPMs in WT mice (42.31 ± 0.62°C) exhibited lower heat thresholds than those in P2Y_1_-/- mice (45.97 ± 0.59°C; p < 0.05; Figure 4B). Similarly, maximum instantaneous frequency during heating was dramatically lower in P2Y_1_-/- mice (2.46 ± 0.35 Hz) versus WT mice (17.77 ± 2.62 Hz; p < 0.05; Figure 4C). The maximal firing rate per degree was also notably higher in WT (6.17 ± 0.53 spikes/deg) than in P2Y_1_-/- mice (1.31 ± 0.14 spikes/deg; p < 0.05). In addition, mean firing rates per degree (44-52°C, p < 0.05) were significantly higher in WT than in P2Y_1_-/- mice (Figure 4D). The heat thresholds of mechanically insensitive CH fibers were unchanged in the P2Y_1_-/- mice (41.8 vs 41.7°C) however, there were only 3 CH fibers characterized in the knockout mice, therefore data was not considered to be statistically valid.

### Immunocytochemical analysis of characterized CPMs

**Figure 5:**
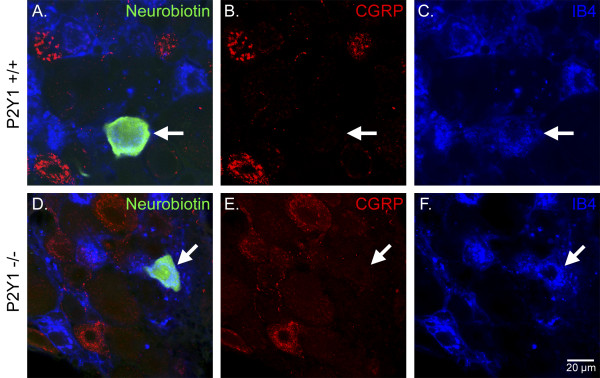
**CPMs in P2Y_1 _transgenic and WT strains bind IB4, but do not express CGRP**. Sample immunohistochemistry of characterized CPM neurons from WT(**A-C**) and P2Y_1_-/- (**D-E**) as indicated by Neurobiotin-labeling (left panels, green). CGRP immunoreactivity (middle panels, red) and labeling with IB4 (right panels, blue) are shown for each strain. Arrows indicate recorded cell. Scale bar represents 20 μm (**F**).

A total of 39 CPM cells (WT: n = 25 cells; and P2Y_1_-/-: n = 14 cells) were labeled with Neurobiotin and subsequently processed for immunohistochemistry. Our findings indicate that the CPMs of P2Y_1_-/- mice express similar patterns of immunohistochemical markers as those contained in WT mice (Figure 5). We found that most Neurobiotin-filled WT CPMs bound IB4 (18/23; cells positive/total cells examined), but did not express either TRPV1 (0/19) or CGRP (0/6). In P2Y_1_-/- mice, Neurobiotin-filled CPMs bound IB4 (11/14), but did not express TRPV1 (0/2) or CGRP (0/12; Figure 5).

### Real-time PCR analysis of P2Y1-/- mice

**Figure 6:**
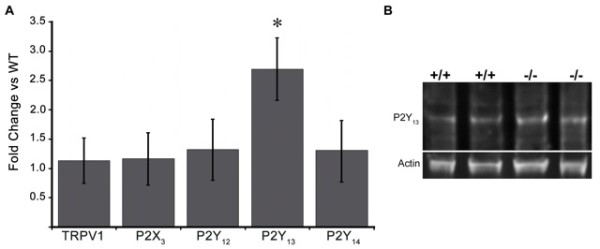
**Changes in gene expression in P2Y1-/- mice**. A. Changes in mRNA levels for TRPV1, P2X_3 _and the Gi-coupled P2Y receptors P2Y_12_, P2Y_13 _and P2Y_14 _in L2-5 DRG. Of the genes examined, only P2Y_13 _levels were significantly altered in P2Y1-/- mice, showing a 2.7-fold increase over wildtype levels (n = 5/genotype, *p < 0.001). B. P2Y13 protein levels were analyzed by multiplex Western blot using actin as a reference standard. Quantification of band density revealed no significant difference in P2Y13 protein levels between WT and P2Y1-/- lumbar DRG (n of 4/genotype; p < 0.97).

A recent study has suggested that P2Y1 and the Gi-coupled P2Y receptors P2Y12-14 interact functionally to modulate nociceptor excitability [21]. To address the possibility that secondary changes in the expression of other receptors may be contribute to the phenotype of P2Y_1_-/- mice, we examined mRNA levels for several genes that could contribute to transduction in cutaneous afferents, including TRPV1, P2X_3 _and the Gi-coupled P2Y receptors, which are likely to be highly-coexpressed with P2Y_1 _and may antagonize excitatory signaling in nociceptors [21]. As expected, expression of TRPV1 and P2X_3 _were unaltered; of the P2Y receptors, P2Y_13 _mRNA levels were significantly increased (Figure 6A, 2.7-fold increase). However, quantitative analysis of protein levels by Western blot indicated no significant difference in the amount of P2Y13 protein between WT and P2Y1-/- DRG (Figure 6B) Therefore, translation of P2Y13 was not altered in the mutant mice.

## Discussion

We provide evidence that P2Y_1 _modulates the transduction of thermal stimuli in cutaneous polymodal nociceptors. P2Y_1 _is highly expressed in DRG neurons and appears to be enriched in IB4-binding neurons, many of which are polymodal nociceptors [31,32]. Functional responses to ADP were widespread in this subset of neurons and were presumably mediated by P2Y_1_, given that ADP-evoked Ca^2+ ^transients are largely absent in neurons from P2Y_1_-/- mice [21]. In mice lacking P2Y_1_, CPM nociceptors showed reduced sensitivity to both heating and cooling, but not mechanical stimuli.

The results presented here demonstrate that cutaneous CPM fibers require P2Y_1 _for the normal transduction of thermal stimuli. However, the underlying mechanism of action of P2Y_1 _remains unclear. P2Y_1 _has been reported to inhibit both Ca^2+^-dependent K^+ ^channels [33] and the M-type K^+ ^current [34] in neurons *in vitro*, which would tend to enhance excitability and firing frequency. Alternatively, it has recently been suggested that G_i_-coupled ADP receptors P2Y_12-13 _inhibit voltage-dependent calcium channels in sensory neurons and this inhibition is enhanced in the absence of P2Y_1 _[21]. However, the finding that the deficit is modality-specific would seem to rule out the possibility that this phenotype is caused by a reduction in overall neuronal excitability.

There are several other possible mechanisms that warrant discussion. First, a growing body of evidence implicates nucleotide signaling in the transduction of noxious stimuli. A recent report demonstrated a graded release of ATP from keratinocytes in response to increasing heat, suggesting that keratinocytes may participate in the transduction of thermal stimuli, particularly in complex with neurons lacking TRPV1 [35]. Keratinocytes, which contain TRPV3 and TRPV4, could respond to heat by releasing ATP and subsequently activating neuronal P2Y_1 _receptors. Nucleotide signaling may thus provide a mechanism for communication from keratinocytes to sensory axon terminals. If this model is correct, then thermal transduction in CPM fibers requires an intact axon-keratinocyte complex and would not be detectable in isolated DRG neurons. This mechanism contrasts with TRPV1-mediated heat responses that can be evoked in isolated neurons *in vitro *[36,37]. Intriguingly, while dissociated neurons isolated from TRPV1 knockout mice showed no heat-gated currents, CPM fibers in TRPV1-/- mice had normal heat responses when in contact with the skin [31,36]. Further support for a role of nucleotide transmission in thermal sensation is provided by several reports suggesting that P2X_3 _contributes to the transduction of warming stimuli [14,38]. It is worth noting that there was no difference in mRNA levels for P2X_3 _between WT and P2Y_1_-/- DRG.

A number of studies have reported that keratinocytes release ATP in response to mechanical as well as thermal stimuli, suggesting a common transduction mechanism for mechanical and heat stimuli in polymodal nociceptors [38]. However, neither P2X_3_-/- [13,14] nor P2Y_1_-/- mice (Figure 2) showed deficits in mechanosensation. Furthermore, we have examined the activation time for these CPM fibers to mechanical stimuli and found that in response to suprathreshold stimuli, action potentials are evoked within a few ms of the onset of the mechanical stimulus. Unfortunately, we are unable to determine the activation time for thermal stimuli, as we cannot deliver thermal stimuli with the same temporal precision as we can with the mechanical stimulus. While it is possible for GPCRs to function directly as stimulus transducers (e.g. rhodopsin) and this process can be quite rapid (e.g. phototransduction), it is difficult to imagine how mechanical stimuli could elicit ATP release from keratinocytes, diffusion of ATP to axon terminals and activation of P2Y signaling in such a rapid fashion. Therefore, a modulatory role is more likely. Two studies have reported a potentiating effect of ATP on mechanically induced signaling in sensory neurons [16,39]. In both studies a role for P2Y_1 _was ruled out by the failure of the P2Y_1_/P2X antagonist PPADS to reverse the ATP effect (note that at publication of the former study it was not yet known that PPADS inhibits P2Y_1_), consistent with the lack of a mechanical phenotype in P2Y_1_-/- cutaneous afferents reported here. Therefore, the actions of P2Y_1 _in CPM stimulus transduction appear to be restricted to thermal stimuli. P2Y receptors likely modulate the function of channels that act as dedicated transducers for thermal stimuli (rev. in [7]). Thus, it is possible that as-yet unidentified ionotropic receptors responsible for the transduction of thermal stimuli in CPM neurons require an interaction with P2Y_1 _receptors for normal function.

### Decreased thermal sensitivity is TRP-independent

In initial studies, Tominaga et al. [40] suggested that P2Y_1 _might modulate TRPV1 function. However, later studies revealed that in sensory neurons P2Y_2 _and not P2Y_1 _is co-expressed with TRPV1 and required for the modulation of TRPV1 by ATP [22,24]. We have previously demonstrated the presence of TRPV1 immunoreactivity in mouse to be exclusively in mechanically-insensitive cutaneous CH afferents [32]. Here as previously, all CPM fibers examined lacked TRPV1 immunoreactivity. In addition, although we recorded from only a few CH fibers (3) in P2Y_1 _deficient mice, there were no apparent effects on their heat sensitivity. Finally, there was no difference in TRPV1 mRNA levels between WT and P2Y_1_-/- DRG.

Our results indicate that P2Y_1 _also contributes to the response of CPM fibers to cold stimuli. Two TRP channels have been implicated in the transduction of cold stimuli: TRPA1 and TRPM8. Although we did not perform immunostaining for TRPA1 or TRPM8, results from previous studies suggest that these channels are not localized in the cutaneous CPM population. For example, TRPM8 is not expressed in IB4-positive neurons [41,42]. Furthermore, G_q_-coupled receptor signaling has been reported to reduce TRPM8 currents [43], whereas in our studies both heat and cold sensitivity were reduced in the absence of P2Y_1_. TRPA1 has also been reported to co-localize almost exclusively with TRPV1 and not with IB4 binding [44,45]. Thus, the impact of P2Y_1 _deletion on the thermal transduction process in cutaneous CPM fibers does not appear to involve any of the known thermosensitive channels responsive to the temperature range examined here.

While the results presented here demonstrate a clear deficit in thermal transduction properties of CPM fibers in mice lacking P2Y_1_, Malin and Molliver [21] reported that these mice had behavioral heat withdrawal latencies that were not different than those of wildtype mice. However, they did find that there was a significant difference in the level of inflammation-induced heat hyperalgesia. While not as striking, this finding is similar to that seen in mice lacking TRPV1, which had relatively normal acute heat responses but did not develop heat hyperalgesia following inflammation [36,37].

It is interesting to note that these two fiber types, CPM and CH fibers, constitute virtually all cutaneous C-fibers that respond to heat. Based on the behavioral studies of these knockout mice it appears that while heat-evoked behaviors can be mediated by both TRPV1-containing CH fibers [46] and TRPV1-deficient CPM fibers [36,37], the relatively small population of CH fibers are more efficient at evoking behavioral responses in mice. This seems to be most clearly evident in the response to heat following inflammation. However, this interpretation may be biased by the fact that while CPM fibers in P2Y1-/- mice show a reduced ability to transduce heat stimuli, TRPV1-/- mice are entirely lacking in CH fibers [32,47]. Thus it is most likely that both afferent populations are necessary for acute heat hyperalgesia following injury.

## Conclusions

It is apparent from the results presented here that P2Y_1 _is necessary for normal thermal (heat and cold) sensitivity in cutaneous CPM fibers. On the other hand, P2Y_1 _is not necessary for normal mechanical sensitivity. This suggests that mechanisms of thermal and mechanical transduction in cutaneous CPM fibers are modulated by separate mechanisms and not merely by control of overall excitability. Consistent with this idea, in transgenic mice overexpressing the neurotrophin GDNF in the skin, we observed an increase in mechanical sensitivity with no change in heat sensitivity [48]. While it is most likely that the effects observed here indicate a modulatory role for P2Y_1 _specific to thermal transduction, further experiments will be required to determine whether P2Y_1 _contributes directly to the transduction of thermal stimuli in the keratinocyte/sensory axon complex.

## Methods

All procedures used in these experiments were reviewed and approved by the Institutional Care and Use Committees at the University of Pittsburgh and followed the guidelines of the International Association for the Study of Pain.

### Mice

Mice with a null mutation in the P2Y_1 _gene have been previously described [49], and were generously provided by Beverly Koller, University of North Carolina, Chapel Hill. These mice thrive and breed normally. The mice were maintained on the C57BL6 background and were genotyped by PCR. Mice were housed in group cages, maintained on a 12:12 hour light-dark cycle in a temperature controlled environment (20.5°C) and given food and water ad libitum.

### Real-Time PCR

Real-time PCR analysis was carried out as previously described [50]. Mice were perfused with ice cold sterile saline. L2-5 dorsal root ganglia were dissected bilaterally and collected in RNAlater solution (Invitrogen). mRNA was isolated using the Qiagen RNeasy Mini kit according to the manufacturer's instructions and quantified by spectrophotometer. Extracted RNA was treated with DNase (Invitrogen) to remove genomic DNA (1 μl DNase, 2 μl 10× DNase buffer, 0.25 μl RNasin/5 μg RNA in H2O, 20 μl total/reaction) and reverse-transcribed using Invitrogen Superscript II reverse transcriptase according to the manufacturer's instructions. Negative control reactions were run without RNA to test for contamination. SYBR Green PCR amplification was performed using an Eppendorf Mastercycler Realplex real-time thermal cycler. All samples were run in triplicate; negative control reactions were run without template and with the reverse-transcriptase negative control reaction products in every amplification run. After amplification, a dissociation curve was plotted against melting temperature to verify selective amplification of a single product. Threshold cycle (Ct) values, the cycle number in which SYBR Green fluorescence rises above background, were recorded as a measure of initial template concentration. Relative changes in RNA levels were calculated by the ΔΔCt method using p53-glyceraldehyde-3-phosphate dehydrogenase (GAPDH) as a reference standard: mean Ct values from each triplicate sample (n = 5 mice/data point, run individually) were subtracted from the mean reference standard Ct, yielding ΔCt. The difference between the ΔCt of the mutant and wildtype groups was then calculated for each gene of interest (ΔΔCt). The relative fold change was determined as 2^-DDCt^. Statistical significance was determined by Student's t-test. Data are presented as the fold change in mRNA levels compared to baseline.

### Calcium Imaging

Dissociation of primary sensory neurons has been described in detail [51]. DRGs from all segmental levels were dissected from adult male WT mice, digested enzymatically and mechanically dissociated by trituration. Ca^2+ ^imaging was performed within 18-24 hours as described previously [22]. Cells were incubated in 2 mM fura-2-AM in HBSS with 5 mg/ml bovine serum albumin and 10 μg/ml IB4 conjugated to Alexa-488 for 30 minutes at 37°C, then mounted on a microscope stage with constantly flowing HBSS at 5 ml/minute. Perfusion rate was controlled with a gravity flow system and perfusate temperature was maintained at 30°C using a heated stage and in-line heating system (Warner Instruments). Drugs were delivered with a rapid-switching local perfusion system. Firmly-attached, refractile cells were identified as regions of interest in the software (Simple PCI, C-Imaging). Absorbance data at 340 and 380 nm were collected once per second and the relative fluorescence (ratio 340/380) was plotted against time. An initial stimulus of buffer with 50 mM K^+ ^was used to confirm the viability and neuronal identity of the cells. Neurons were then tested for responsiveness to the P2X agonist α,β-methylene ATP (a,b-me ATP) and ADP. Ca^2+ ^transients were examined in response to application of agonists as noted in the figure legend.

### Ex Vivo Preparation

The *ex vivo *somatosensory system preparation has been previously described in detail [52,53]. Briefly, adult C57BL6 mice (Jackson Laboratory, Bar Harbor, ME) and P2Y_1 _transgenic mice were anesthetized via an intramuscular injection of ketamine and xylazine (90 and 10 mg/kg, respectively) and perfused transcardially with chilled (6°C), oxygenated (95% O_2_-5% CO_2_) artificial CSF (aCSF; in mmol/l: 1.9 KCl, 1.2 KH_2_PO_4_, 1.3 MgSO_4_, 2.4 CaCl_2_, 26.0 NaHCO_3_, and 10.0 D-glucose), with 253.9 mmol/l sucrose. Spinal cord, L1-L4 DRGs, saphenous nerve, and innervated skin were dissected free in continuity. Following dissection, the preparation was transferred to a separate recording chamber containing chilled oxygenated aCSF in which the sucrose was replaced with 127.0 mmol/l NaCl. The skin was pinned out on a stainless steel grid located at the bath/air interface, so that the dermal surface remained perfused with the aCSF while the epidermis was exposed to the air. The platform provided stability during the application of thermal and mechanical stimuli. The bath was then slowly warmed to 31°C prior to recording.

### Recording and Stimulation

Individual DRG cells were impaled with quartz filament microelectrodes (impedance >100 MΩ) containing 5% Neurobiotin (Vector Laboratories, Burlingame, CA) in 1 mol/l potassium acetate. Electrical search stimuli were delivered through a suction electrode on the saphenous nerve to locate sensory neuron somata with a peripheral axon innervating the skin. Peripheral receptive fields (RF) were localized with a fine paint brush, blunt glass probe and von Frey hairs. When cells were driven by the nerve, but had no mechanical RF, a thermal search was performed by applying hot (~52°C) or cold (~0°C) physiological saline to the skin using a 10 ml syringe with a 20-gauge needle. If a thermal RF was located, the absence of mechanical sensitivity was confirmed by searching the identified RF using a glass probe. The response characteristics of the sensory neuron were determined by applying computer controlled mechanical and thermal stimuli. The mechanical stimulator consisted of a constant force controller (Aurora Scientific Aurora, Ontario, Canada) attached to a 1 mm diameter plastic disc. Computer controlled 5 s square waves of 5, 10, 25, 50, and 100 mN were applied to the cell's RF. Mechanical threshold was the lowest stimulus intensity of this ascending series to elicit at least one action potential (AP) within the first second of stimulus application. After mechanical stimulation, thermal stimuli were applied using a 3 mm^2 ^contact area Peltier element (Yale University Machine Shop). The cooling stimulus was rapidly applied by the Peltier element through the thermal conduction of circulating ice-chilled water that resulted in a drop in temperature from 31°C to approximately 4-6°C. The temperature was then brought back up to 31°C, and after a 5 s pause the heating stimulus was applied, consisting of a 12 s heat ramp from 31°C to 52°C followed by a 5 s plateau at 52°C. The stimulus then ramped back down to 31°C in 12 seconds. The cooling and heating thermal thresholds were determined to be the temperatures at which the first AP was evoked. All responses were recorded digitally for off-line analysis (Spike2 software; Cambridge Electronic Design, Cambridge, UK). After physiological characterization, the cell was labeled by iontophoretically injecting Neurobiotin (1-3 cells per DRG). Peripheral conduction velocity was calculated from spike latency and the distance between the stimulating and recording electrodes.

### Tissue Processing and Analysis of Recorded Cells

Once a sensory neuron was characterized and filled with Neurobiotin, the DRG containing the injected cell was removed and immersion fixed (4% paraformaldehyde in 0.1 M phosphate buffer for 30 minutes at 4°C). Ganglia were then embedded and blocked in 10% gelatin, postfixed overnight, and cryoprotected in 20% sucrose. Frozen sections (60 μm) were collected in phosphate buffer and reacted with antiserum for either TRPV1 (rabbit anti-TRPV1; Calbiochem, San Diego, CA) or CGRP (rabbit anti-CGRP; Chemicon, Temecula, CA). The binding of isolectin B_4 _from *Griffonia simplicifolia *was examined using IB4-647 (Molecular Probes, Eugene, OR). After incubation in primary antiserum, tissue was washed and incubated in donkey anti-rabbit secondary antiserum conjugated to Cy3 (Jackson Immunoresearch, West Grove, PA), and reacted with FITC-conjugated avidin to label Neurobiotin-filled cells (Vector Laboratories). Distribution of fluorescent staining was determined using an Olympus confocal microscope and software (Fluoview; Olympus, La Jolla, CA). Sequential scanning was done to prevent bleed-through of the different fluorophores.

### Multiplex Western Blotting

Lumbar DRG (L2 - L5) were homogenized in lysis buffer containing 25 mM Tris-HCl pH 7.4, 150 mM NaCl, 1 mM EDTA, 1% NP-40 and 5% glycerol. Lysates containing equal amounts of protein per 30 μl for each sample were heated at 60°C for 5 min in 9% SDS, 60% glycerol, 375 mM Tris-HCl pH and bromophenol blue 0.015%, centrifuged and resolved by sodium dodecyl sulfate-polyacrylamide gel electrophoresis (SDS-PAGE) followed by transfer onto nitrocellulose membranes. Membranes were blocked in 5% bovine serum albumin in PBS with 0.05% Tween 20 (PBS-T) and incubated overnight at 4°C with both rabbit anti-P2Y_13 _antibody (1:500) and mouse anti-actin (1:500). The actin monoclonal antibody, developed by Dr. Jim Jung-Chin Lin, was obtained from the Developmental Studies Hybridoma Bank developed under the auspices of the NICHD and maintained by The University of Iowa, Department of Biology, Iowa City, IA 52242. Primary antibodies were followed by detection with SpectraPlex™ Fluorescent Western Blot secondary antibodies: APC-goat anti-rabbit IgG (for P2Y13) and donkey anti-mouse conjugated to CY3 at 1: 2500 dilution for both. Blots were imaged using a FluorChem Q workstation (Cell Biosciences, Santa Clara, CA). P2Y_13 _band density normalized to actin (as a loading control) was quantified using the manufacturer's software (n = 4/genotype).

### Data Analysis

Data are expressed as means ± SE. Unpaired two-tailed Student's *t*-tests were used to analyze different aspects of the *ex vivo *responses of neurons to electrical, mechanical and heat stimuli. Heat data was normalized by multiplying the average AP spikes per degree by the percentage of cells responding at that temperature. In the analysis of mean firing rate/°C in response to the application of the heat ramp a 2-way ANOVA analysis was completed and followed with Bonferroni post hoc analysis. Statistical analysis for the real-time PCR results was performed on the raw Ct data and presented as fold changes in mRNA levels for clarity.

## Competing interests

The authors declare that they have no competing interests.

## Authors' contributions

DCM, designed and conducted calcium imaging, PCR experiments and Western blots and data analysis and wrote manuscript. KKR conducted *ex vivo *recording experiments performed data analysis and wrote manuscript. SLM helped conduct *ex vivo *experiments, MPJ, helped conduct *ex vivo *experiments and helped with data analysis and contributed to writing the manuscript. HRK designed the study and helped conduct *ex vivo *experiments and wrote manuscript.

All authors have read and approved the final manuscript.

## References

[B1] Burnstock G Current status of purinergic signalling in the nervous system Prog Brain Res 1999 120 3 10 full_text 10550983 10.1016/s0079-6123(08)63541-4

[B2] Hamilton SG Warburton J Bhattacharjee A Ward J McMahon SB ATP in human skin elicits a dose-related pain response which is potentiated under conditions of hyperalgesia Brain 2000 123 Pt 6 1238 1246 10.1093/brain/123.6.1238 10825361

[B3] Bland-Ward PA Humphrey PP Acute nociception mediated by hindpaw P2X receptor activation in the rat Br J Pharmacol 1997 122 365 371 10.1038/sj.bjp.0701371 9313948 PMC1564928

[B4] Ralevic V Burnstock G Receptors for purines and pyrimidines Pharmacol Rev 1998 50 413 492 9755289

[B5] Boeynaems JM Communi D Gonzalez NS Robaye B Overview of the P2 receptors Semin Thromb Hemost 2005 31 139 149 10.1055/s-2005-869519 15852217

[B6] Hussl S Boehm S Functions of neuronal P2Y receptors Pflugers Arch 2006 452 538 551 10.1007/s00424-006-0063-8 16691392

[B7] Volonte C Amadio S D'Ambrosi N Colpi M Burnstock G P2 receptor web: complexity and fine-tuning Pharmacol Ther 2006 112 264 280 10.1016/j.pharmthera.2005.04.012 16780954

[B8] Burnstock G Purine and pyrimidine receptors Cell Mol Life Sci 2007 64 1471 1483 10.1007/s00018-007-6497-0 17375261 PMC11149472

[B9] Donnelly-Roberts D McGaraughty S Shieh CC Honore P Jarvis MF Painful purinergic receptors J Pharmacol Exp Ther 2008 324 409 415 10.1124/jpet.106.105890 18042830

[B10] Chen CC Akopian AN Sivilotti L Colquhoun D Burnstock G Wood JN A P2X purinoceptor expressed by a subset of sensory neurons Nature 1995 377 428 431 10.1038/377428a0 7566119

[B11] Lewis C Neidhart S Holy C North RA Buell G Surprenant A Coexpression of P2X2 and P2X3 receptor subunits can account for ATP-gated currents in sensory neurons Nature 1995 377 432 435 10.1038/377432a0 7566120

[B12] Bradbury EJ Burnstock G McMahon SB The Expression of P2X3 Purinoreceptors in Sensory Neurons: Effects of Axotomy and Glial-Derived Neurotrophic Factor Mol Cell Neurosci 1998 12 256 268 10.1006/mcne.1998.0719 9828090

[B13] Cockayne DA Hamilton SG Zhu QM Dunn PM Zhong Y Novakovic S Malmberg AB Cain G Berson A Kassotakis L Urinary bladder hyporeflexia and reduced pain-related behaviour in P2X3-deficient mice Nature 2000 407 1011 1015 10.1038/35039519 11069181

[B14] Souslova V Cesare P Ding Y Akopian AN Stanfa L Suzuki R Carpenter K Dickenson A Boyce S Hill R Warm-coding deficits and aberrant inflammatory pain in mice lacking P2X3 receptors Nature 2000 407 1015 1017 10.1038/35039526 11069182

[B15] Cockayne DA Dunn PM Zhong Y Rong W Hamilton SG Knight GE Ruan HZ Ma B Yip P Nunn P P2X2 knockout mice and P2X2/P2X3 double knockout mice reveal a role for the P2X2 receptor subunit in mediating multiple sensory effects of ATP J Physiol 2005 567 621 639 10.1113/jphysiol.2005.088435 15961431 PMC1474198

[B16] Nakamura F Strittmatter SM P2Y1 purinergic receptors in sensory neurons: contribution to touch-induced impulse generation Proc Natl Acad Sci USA 1996 93 10465 10470 10.1073/pnas.93.19.10465 8816824 PMC38408

[B17] Molliver DC Cook SP Carlsten JA Wright DE McCleskey EW ATP and UTP excite sensory neurons and induce CREB phosphorylation through the metabotropic receptor, P2Y2 Eur J Neurosci 2002 16 1850 1860 10.1046/j.1460-9568.2002.02253.x 12453048

[B18] Sanada M Yasuda H Omatsu-Kanbe M Sango K Isono T Matsuura H Kikkawa R Increase in intracellular Ca(2+) and calcitonin gene-related peptide release through metabotropic P2Y receptors in rat dorsal root ganglion neurons Neuroscience 2002 111 413 422 10.1016/S0306-4522(02)00005-2 11983326

[B19] Ruan HZ Burnstock G Localisation of P2Y1 and P2Y4 receptors in dorsal root, nodose and trigeminal ganglia of the rat Histochem Cell Biol 2003 120 415 426 10.1007/s00418-003-0579-3 14564529

[B20] Kobayashi K Fukuoka T Yamanaka H Dai Y Obata K Tokunaga A Noguchi K Neurons and glial cells differentially express P2Y receptor mRNAs in the rat dorsal root ganglion and spinal cord J Comp Neurol 2006 498 443 454 10.1002/cne.21066 16874807

[B21] Malin SA Molliver DC Gi- and Gq-coupled ADP (P2Y) receptors act in opposition to modulate nociceptive signaling and inflammatory pain behavior Mol Pain 2010 6 21 10.1186/1744-8069-6-21 20398327 PMC2865444

[B22] Malin SA Davis BM Koerber HR Reynolds IJ Albers KM Molliver DC Thermal nociception and TRPV1 function are attenuated in mice lacking the nucleotide receptor P2Y2 Pain 2008 138 484 496 10.1016/j.pain.2008.01.026 18343036 PMC2630699

[B23] Chen X Molliver DC Gebhart GF The P2Y2 receptor sensitizes mouse bladder sensory neurons and facilitates purinergic currents J Neurosci 2010 30 2365 2372 10.1523/JNEUROSCI.5462-09.2010 20147562 PMC2828760

[B24] Moriyama T Iida T Kobayashi K Higashi T Fukuoka T Tsumura H Leon C Suzuki N Inoue K Gachet C Possible involvement of P2Y2 metabotropic receptors in ATP-induced transient receptor potential vanilloid receptor 1-mediated thermal hypersensitivity J Neurosci 2003 23 6058 6062 12853424 10.1523/JNEUROSCI.23-14-06058.2003 PMC6740351

[B25] Gerevich Z Borvendeg SJ Schroder W Franke H Wirkner K Norenberg W Furst S Gillen C Illes P Inhibition of N-type voltage-activated calcium channels in rat dorsal root ganglion neurons by P2Y receptors is a possible mechanism of ADP-induced analgesia J Neurosci 2004 24 797 807 10.1523/JNEUROSCI.4019-03.2004 14749424 PMC6729814

[B26] Gerevich Z Muller C Illes P Metabotropic P2Y1 receptors inhibit P2X3 receptor-channels in rat dorsal root ganglion neurons Eur J Pharmacol 2005 521 34 38 10.1016/j.ejphar.2005.08.001 16181623

[B27] Chen Y Zhang X Wang C Li G Gu Y Huang LY Activation of P2X7 receptors in glial satellite cells reduces pain through downregulation of P2X3 receptors in nociceptive neurons Proc Natl Acad Sci USA 2008 105 16773 16778 10.1073/pnas.0801793105 18946042 PMC2575495

[B28] Borvendeg SJ Gerevich Z Gillen C Illes P P2Y receptor-mediated inhibition of voltage-dependent Ca2+ channels in rat dorsal root ganglion neurons Synapse 2003 47 159 161 10.1002/syn.10156 12454954

[B29] Kress M Koltzenburg M Reeh PW Handwerker HO Responsiveness and functional attributes of electrically localized terminals of cutaneous C-fibers in vivo and in vitro J Neurophysiol 1992 68 581 595 1527577 10.1152/jn.1992.68.2.581

[B30] Koltzenburg M Stucky CL Lewin GR Receptive properties of mouse sensory neurons innervating hairy skin J Neurophysiol 1997 78 1841 1850 9325353 10.1152/jn.1997.78.4.1841

[B31] Woodbury CJ Zwick M Wang S Lawson JJ Caterina MJ Koltzenburg M Albers KM Koerber HR Davis BM Nociceptors lacking TRPV1 and TRPV2 have normal heat responses J Neurosci 2004 24 6410 6415 10.1523/JNEUROSCI.1421-04.2004 15254097 PMC6729548

[B32] Lawson JJ McIlwrath SL Woodbury CJ Davis BM Koerber HR TRPV1 unlike TRPV2 is restricted to a subset of mechanically insensitive cutaneous nociceptors responding to heat J Pain 2008 9 298 308 10.1016/j.jpain.2007.12.001 18226966 PMC2372162

[B33] Schicker KW Chandaka GK Geier P Kubista H Boehm S P2Y1 receptors mediate an activation of neuronal calcium-dependent K+ channels J Physiol 2010 in press 20679351 10.1113/jphysiol.2010.193367 PMC2998222

[B34] Filippov AK Choi RC Simon J Barnard EA Brown DA Activation of P2Y1 nucleotide receptors induces inhibition of the M-type K+ current in rat hippocampal pyramidal neurons J Neurosci 2006 26 9340 9348 10.1523/JNEUROSCI.2635-06.2006 16957090 PMC1855006

[B35] Mandadi S Sokabe T Shibasaki K Katanosaka K Mizuno A Moqrich A Patapoutian A Fukumi-Tominaga T Mizumura K Tominaga M TRPV3 in keratinocytes transmits temperature information to sensory neurons via ATP Pflugers Arch 2009 458 1093 1102 10.1007/s00424-009-0703-x 19669158 PMC2745623

[B36] Caterina MJ Leffler A Malmberg AB Martin WJ Trafton J Petersen-Zeitz KR Koltzenburg M Basbaum AI Julius D Impaired nociception and pain sensation in mice lacking the capsaicin receptor Science 2000 288 306 313 10.1126/science.288.5464.306 10764638

[B37] Davis JB Gray J Gunthorpe MJ Hatcher JP Davey PT Overend P Harries MH Latcham J Clapham C Atkinson K Vanilloid receptor-1 is essential for inflammatory thermal hyperalgesia Nature 2000 405 183 187 10.1038/35012076 10821274

[B38] Dussor G Koerber HR Oaklander AL Rice FL Molliver DC Nucleotide signaling and cutaneous mechanisms of pain transduction Brain Res Rev 2009 60 24 35 10.1016/j.brainresrev.2008.12.013 19171165 PMC3201739

[B39] Lechner SG Lewin GR Peripheral sensitisation of nociceptors via G-protein-dependent potentiation of mechanotransduction currents J Physiol 2009 587 3493 3503 10.1113/jphysiol.2009.175059 19505980 PMC2742277

[B40] Tominaga M Wada M Masu M Potentiation of capsaicin receptor activity by metabotropic ATP receptors as a possible mechanism for ATP-evoked pain and hyperalgesia Proc Natl Acad Sci USA 2001 98 6951 6956 10.1073/pnas.111025298 11371611 PMC34459

[B41] Peier AM Moqrich A Hergarden AC Reeve AJ Andersson DA Story GM Earley TJ Dragoni I McIntyre P Bevan S Patapoutian A A TRP channel that senses cold stimuli and menthol Cell 2002 108 705 715 10.1016/S0092-8674(02)00652-9 11893340

[B42] Dhaka A Earley TJ Watson J Patapoutian A Visualizing cold spots: TRPM8-expressing sensory neurons and their projections J Neurosci 2008 28 566 575 10.1523/JNEUROSCI.3976-07.2008 18199758 PMC6670358

[B43] Premkumar LS Raisinghani M Pingle SC Long C Pimentel F Downregulation of transient receptor potential melastatin 8 by protein kinase C-mediated dephosphorylation J Neurosci 2005 25 11322 11329 10.1523/JNEUROSCI.3006-05.2005 16339027 PMC6725906

[B44] Bautista DM Movahed P Hinman A Axelsson HE Sterner O Hogestatt ED Julius D Jordt SE Zygmunt PM Pungent products from garlic activate the sensory ion channel TRPA1 Proc Natl Acad Sci USA 2005 102 12248 12252 10.1073/pnas.0505356102 16103371 PMC1189336

[B45] Kobayashi K Fukuoka T Obata K Yamanaka H Dai Y Tokunaga A Noguchi K Distinct expression of TRPM8, TRPA1, and TRPV1 mRNAs in rat primary afferent neurons with adelta/c-fibers and colocalization with trk receptors J Comp Neurol 2005 493 596 606 10.1002/cne.20794 16304633

[B46] Cavanaugh DJ Lee H Lo L Shields SD Zylka MJ Basbaum AI Anderson DJ Distinct subsets of unmyelinated primary sensory fibers mediate behavioral responses to noxious thermal and mechanical stimuli Proc Natl Acad Sci USA 2009 106 9075 9080 10.1073/pnas.0901507106 19451647 PMC2683885

[B47] Koerber HR McIlwrath SL Lawson JJ Malin SA Anderson CE Jankowski MP Davis BM Cutaneous C-polymodal fibers lacking TRPV1 are sensitized to heat following inflammation, but fail to drive heat hyperalgesia in the absence of TRPV1 containing C-heat fibers Mol Pain 2010 6 58 10.1186/1744-8069-6-58 20858240 PMC2949725

[B48] Albers KM Woodbury CJ Ritter AM Davis BM Koerber HR Glial cell-line-derived neurotrophic factor expression in skin alters the mechanical sensitivity of cutaneous nociceptors J Neurosci 2006 26 2981 2990 10.1523/JNEUROSCI.4863-05.2006 16540576 PMC6673969

[B49] Fabre JE Nguyen M Latour A Keifer JA Audoly LP Coffman TM Koller BH Decreased platelet aggregation, increased bleeding time and resistance to thromboembolism in P2Y1-deficient mice Nat Med 1999 5 1199 1202 10.1038/13522 10502826

[B50] Molliver DC Lindsay J Albers KM Davis BM Overexpression of NGF or GDNF alters transcriptional plasticity evoked by inflammation Pain 2005 113 277 284 10.1016/j.pain.2004.10.025 15661434

[B51] Malin SA Davis BM Molliver DC Production of dissociated sensory neuron cultures and considerations for their use in studying neuronal function and plasticity Nat Protoc 2007 2 152 160 10.1038/nprot.2006.461 17401349

[B52] Woodbury CJ Ritter AM Koerber HR Central anatomy of individual rapidly adapting low-threshold mechanoreceptors innervating the "hairy" skin of newborn mice: early maturation of hair follicle afferents J Comp Neurol 2001 436 304 323 10.1002/cne.1069 11438932

[B53] McIlwrath SL Lawson JJ Anderson CE Albers KM Koerber HR Overexpression of neurotrophin-3 enhances the mechanical response properties of slowly adapting type 1 afferents and myelinated nociceptors Eur J Neurosci 2007 26 1801 1812 10.1111/j.1460-9568.2007.05821.x 17897394

